# Evaluation of efficacy and optimal therapeutic parameters of ALA-PDT in the management of HPV-associated cervical intraepithelial neoplasia grade I

**DOI:** 10.3389/fonc.2026.1889112

**Published:** 2026-07-10

**Authors:** Qian Yu, Ling Wei, Lirui Wu, Suning Chen, Bo Liu, Lianlian Gao

**Affiliations:** Department of Gynecology, Hebei Central Hospital of Petrochina, Hebei, China

**Keywords:** ALA-PDT, CIN I, efficacy analysis, HPV infection, optimal therapeutic parameters, photodynamic therapy

## Abstract

**Objective:**

This study aimed to address three clinically relevant questions in the management of HPV-associated cervical intraepithelial neoplasia grade I (CIN I): whether aminolevulinic acid photodynamic therapy (ALA-PDT) is effective and safe for CIN I, which combination of ALA application time and light exposure duration provides the optimal therapeutic effect, and whether specific HPV subtypes are associated with treatment outcomes.

**Methods:**

We enrolled 240 patients diagnosed with CIN I via colposcopy and pathology from June 2021 to June 2023. Patients were divided into four groups based on ALA application time and light exposure duration: Group A1 (3-hour application, 30-minute light exposure), Group A2 (3-hour application, 40-minute light exposure), Group B1 (5-hour application, 30-minute light exposure), and Group B2 (5-hour application, 40-minute light exposure), with 60 patients per group. A 20% ALA solution was used, and irradiation was performed with 635nm red light. Treatment was administered every 2 weeks for four sessions. Patients were followed for 12 months after treatment. HPV testing, TCT, and colposcopy were performed at 3 and 6 months to assess treatment response, while recurrence, progression, and safety outcomes were monitored throughout the 12-month follow-up period. Cure was defined as the absence of abnormalities in TCT, colposcopic findings, and pathological CIN 6 months post-treatment.

**Results:**

At 3 and 6 months post-treatment, HPV positivity rates decreased and negative conversion rates increased significantly across all groups, with the greatest improvements observed in Group B2 (P<0.05). Additionally, the proportions of HPV16 and HPV18 types decreased significantly in all groups, with the largest reduction in Group B2. Other high-risk HPV types, such as HPV31 and HPV33, also showed significant reductions (P<0.05). Group B2 had the highest cure rate and lowest recurrence rate among all groups (P<0.05). No significant differences were observed in progression rates or incidence of adverse reactions across groups (P>0.05). HPV16 and HPV18 were positively correlated with the cure rate (correlation coefficients of 0.432 and 0.421, respectively; P<0.05).

**Conclusion:**

ALA-PDT is effective for treating HPV-associated CIN I, with optimal parameters of a 5-hour ALA application and 40-minute light exposure.

## 1 Introduction

Cervical intraepithelial neoplasia (CIN) is a group of cervical squamous intraepithelial dysplasia closely related to cervical invasive carcinoma (1). The occurrence of CIN is closely related to human papillomavirus (HPV) infection, especially the infection rate of high-risk HPV is more than 90% in CIN patients, among which the most common subtypes are HPV16 and HPV18 (2). In addition to HPV infection, the occurrence of CIN is also related to a variety of factors, including smoking, aging, multiple sexual partners, history of other HPV-associated lower reproductive tract diseases, history of hysterectomy, history of radiation therapy, immunosuppression, and history of metristilbestrol exposure, etc. (3–5).

At present, the treatment methods of CIN mainly include local drug therapy (such as 5-fluorouracil and 5% imiquimote), carbon dioxide laser vaporization, laser excision, surgical excision and vaginal brachytherapy. Although these treatments are effective to a certain extent, each has certain limitations (6, 7). The effect of local drug therapy is not ideal, and laser vaporization therapy has high requirements for technology and equipment (8). Although surgical resection has a higher cure rate and a lower recurrence rate, it has more complications, such as bleeding, inflammation, poor healing and scar formation, and can cause tissue defects. About 57% of patients with CIN Grade I resolve naturally, but 43% of cases may persist or progress. Therefore, it is particularly important to select a treatment with less damage to the body and better efficacy (9).

Photodynamic therapy (PDT) is a minimally invasive treatment that combines a photosensitizer with a specific wavelength of light. The photosensitizer selectively accumulates in the lesion area through local or systemic administration, and illumination with a specific wavelength induces photochemical reactions that lead to apoptosis or necrosis of diseased cells, thereby achieving therapeutic effects. Studies have confirmed that PDT has significant efficacy in the treatment of persistent high-risk HPV infection of the female lower genital tract, genital condyloma acuminatum, CIN, and other diseases (10). Recent clinical studies have further supported the application of ALA-PDT in HPV-associated cervical lesions. Gu et al. reported that local ALA-PDT was effective for cervical low-grade squamous intraepithelial lesions with high-risk HPV infection, with favorable pathological remission and HPV clearance outcomes (11). Li et al. found that 5-ALA-PDT achieved high histological regression and HR-HPV clearance rates in patients with cervical LSIL, especially in those requiring fertility-preserving treatment (12). In addition, Han et al. reported that 5-ALA-PDT was effective and safe for cervical and vaginal intraepithelial neoplasia and contributed to HPV clearance with minimal adverse effects (13). However, at present, the specific therapeutic parameters of PDT in the treatment of CIN I, such as application time and irradiation time, have not been clearly defined, and the correlation between its efficacy and specific HPV types has not been thoroughly studied. This study aims to explore the efficacy and safety of different treatment parameters, determine the optimal combination of treatment time, and analyze the correlation between HPV typing and treatment outcomes, in order to provide clinical reference.

## 2 Data and methods

### 2.1 General information

A total of 240 consecutive patients diagnosed with cervical intraepithelial neoplasia grade I (CIN I) by colposcopy and pathological examination in the Department of Obstetrics and Gynecology of our hospital between June 2021 and June 2023 were enrolled. According to the treatment parameter combinations, patients were allocated into four groups of 60 cases each: Group A1 (3-hour ALA application and 30-minute light exposure), Group A2 (3-hour ALA application and 40-minute light exposure), Group B1 (5-hour ALA application and 30-minute light exposure), and Group B2 (5-hour ALA application and 40-minute light exposure). Inclusion criteria: Pathological diagnosis was CIN I grade; The patient had not received any other treatment before treatment; Patients ranged in age from 18 to 60; Patients with HPV infection; Patient informed consent. Exclusion criteria: Pregnant and lactating patients; Patients with severe heart, liver and renal insufficiency; A history of radiation therapy;

Patients who are allergic to porphyrins or have a history of photosensitivity; Patients with gonorrhea and non-gonococcal acute infection of urethra and lower reproductive tract were excluded before treatment. Patients who were unable to complete the course of treatment and were followed for 12 months.

**Figure 1:**
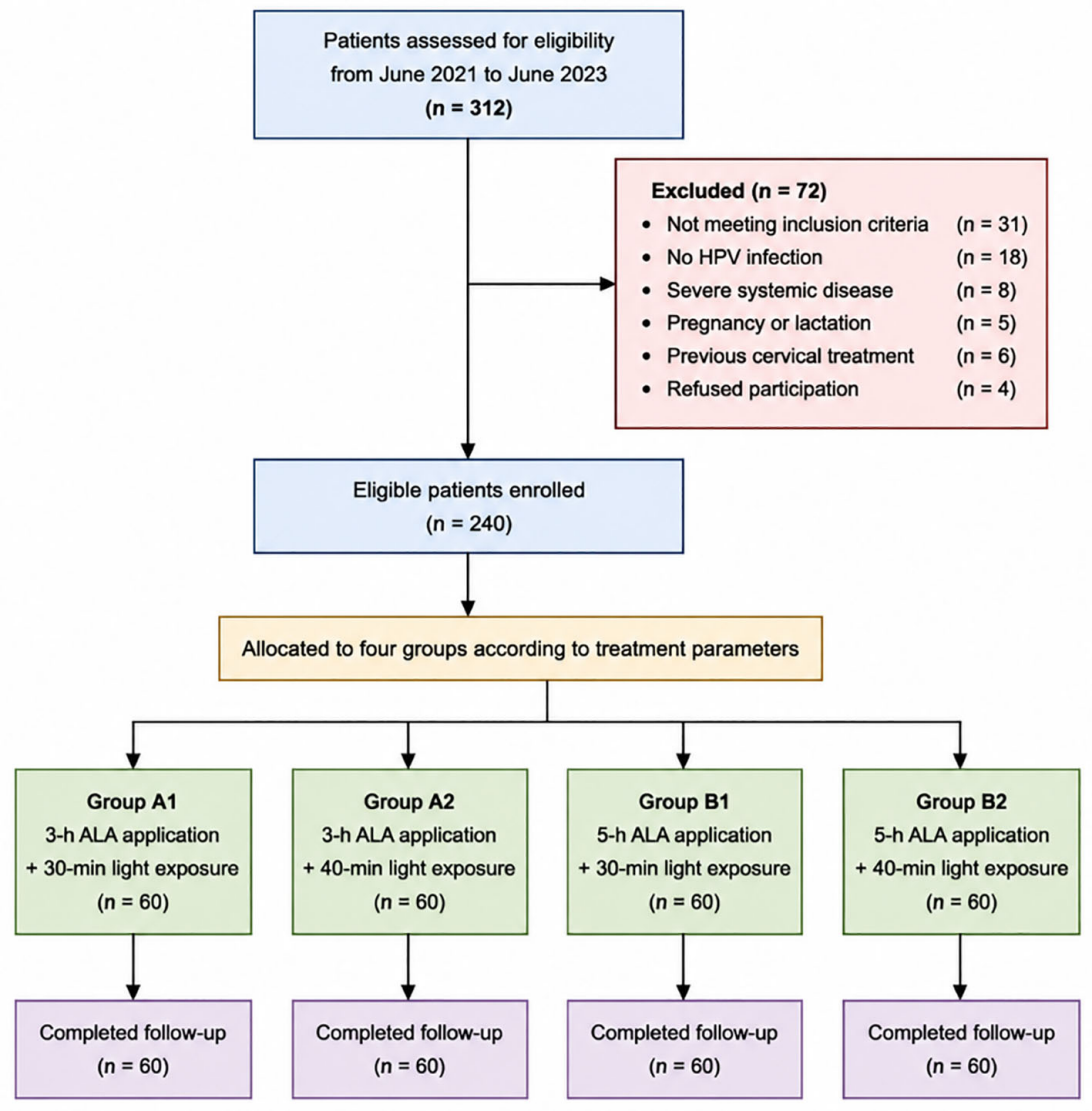
Flowchart of patient selection, enrollment, allocation, and follow-up.

This study was approved by the Ethics Committee of Hebei Central Hospital of PetroChina (Approval Number: HCHP-2021-042, Approval Date: 15 May 2021). All participants provided written informed consent prior to enrollment. All procedures were conducted in accordance with the Declaration of Helsinki and relevant national guidelines. The study overview flowchart is shown in **Figure 1**.

### 2.2 Methods

HPV was detected by inserting a special HPV sampling brush into the cervix, rotating it clockwise/counterclockwise for 4–5 weeks and remaining for 10s, placing it into a vial containing preservation solution, breaking the brush handle, capping the bottle and sending it for examination. hybrid capture 2, a hybrid capture 2 method provided by Digene, was adopted. HC2) detected the content of 13 high-risk HPV-DNA (HPV16, 18, 31, 33, 35, 39, 45, 51, 52, 56, 58, 59, 68) and 5 low-risk HPV-DNA (HPV6, 11, 42, 43, 44) in the samples. The detection result was expressed by the ratio of relative light unit (RIU) to cut off (CO), and RIU/CO≥1.0 was positive.

Ala-pdt is treated with aminolevulinic acid (ALA) (aminovaleric acid hydrochloride external powder, 118mg/branch, Sinopath approval number H20070027, Shanghai Fudan Zhangjiang Bio-Pharmaceutical Co., LTD.) as a photosensitizer, and the photodynamic therapeutic instrument uses LED-IB, from Wuhan YAG Photoelectric Technology Co., LTD. The specific steps are as follows:

Preparation: Do not drink water in the morning of the day of treatment, and ask the patient to empty urine before treatment. The patient was placed on the gynecological examination table with lithotomy, and the location of the lesion was determined with the assistance of colposcope. Images were taken with colposcope.

Dispensing: ALA was prepared into 20% concentration solution by extracting temperature-sensitive gel solvent before clinical use. For each treatment, the liquid medicine must be prepared fresh and stored for no more than 4 hours.

Applying medicine: the affected area is cleaned, and the prepared medicine drops are placed on the cotton ball to cover the lesion and 2cm outside the edge. The continuous applying time is carried out according to groups.

Illumination: 635nm red light is directly irradiated to the patient’s lesions, the energy density is 80–100 J/cm2, the treatment spot should completely cover the lesion, and the irradiation time is according to the group. Treatment was given once every 2 weeks for 4 times.

Patients were followed for 12 months after completion of treatment. HPV testing, cervical cytology (TCT), and colposcopic examinations were performed at 3 and 6 months to evaluate treatment response. Continued follow-up until 12 months was conducted to monitor disease recurrence, progression, and treatment-related adverse events. Histopathological examination was performed when clinically indicated. The standard of cure was 6 months after treatment, when TCT results showed no abnormal squamous cells, colposcopy showed no obvious lesions, and histological diagnosis showed no CIN, no matter whether the HPV test result was positive or not, it was considered to be cured. The criteria for recurrence were no lesions within 6 months of follow-up, colposcopy at follow-up 6 months later indicated obvious lesions, and histological diagnosis was CIN by biopsy. The criteria for progression were elevated CIN grade confirmed by histology and pathology during follow-up, or progression to cervical cancer.

### 2.3 Observation indicators

The primary endpoint of this study was the clinical cure rate at 6 months after treatment. Secondary endpoints included HPV positivity rate, HPV negative conversion rate, changes in HPV subtype distribution, recurrence rate, progression rate, and treatment-related adverse events.

HPV test results were collected before treatment and at 3 and 6 months after treatment, and the changes of HPV positive rate and negative conversion rate were calculated. The results of HPV typing before treatment and 3 and 6 months after treatment were recorded and calculated, paying special attention to the proportion of high-risk HPV subtypes (HPV16, HPV18, HPV31, HPV33, etc.) in each group. The number changes of different HPV subtypes before and after treatment were analyzed to evaluate the clearance effect of ALA-PDT treatment on different HPV subtypes. Clinical efficacy evaluation mainly includes cure rate, recurrence rate and progression rate. Cure rate refers to 6 months after treatment, cervical cytology (TCT) results no abnormal squamous cells, colposcopy no obvious lesions, histopathology no CIN presence, regardless of whether the HPV test result is positive or not is considered to be cured. Recurrence rate refers to the absence of lesions within 6 months of follow-up, but after 6 months of follow-up, colposcopy revealed obvious lesions and histopathology confirmed CIN. Progression rate refers to histopathological evidence of elevated CIN grade or progression to cervical cancer during follow-up. Secondary outcome measures in this study were treatment-related adverse reactions and the association between HPV typing and clinical efficacy. Adverse reactions were recorded and analyzed during and after treatment, including increased vaginal discharge, spotty bleeding, local pruritus discomfort, and cervical stenosis. The incidence of adverse reactions in different groups of patients was analyzed, the effects of different therapeutic parameters on adverse reactions were compared, and the safety of ALA-PDT treatment was evaluated. In addition, correlations were calculated based on HPV typing changes and clinical efficacy outcomes.

### 2.4 Statistical analysis

SPSS 22.0 statistical software was used to process all data. The measurement data were represented by mean ± standard deviation. One-way ANOVA was used for comparison between groups conforming to normal distribution and with homogeneity of variance, and LSD method was used for pairwise comparison if differences existed. The rank sum test was used for comparison between groups that did not conform to normal distribution and homogeneity of variance, and the Kruskal-Wallis method was used for pair comparison when differences existed. Use cases and percentages of count data were expressed, and chi-square tests were compared between independent sample groups. Correlation analysis was performed by Pearson correlation analysis, and P < 0.05 was considered statistically significant. The sample size was determined based on feasibility considerations and the expected differences in clinical cure rates among treatment groups. A total sample size of 240 patients (60 patients per group) was considered sufficient to provide adequate statistical power for detecting clinically meaningful differences in efficacy outcomes while maintaining balanced group sizes for comparative analyses.

## 3 Results

### 3.1 Baseline characteristics of patients

**Table 1** shows baseline characteristics of patients in the four groups, including age, BMI, smoking history, other HPV-related medical history, and history of hysterectomy. The results showed that there was no statistical significance in baseline characteristics among the four groups (P>0/05), and there was comparability between the groups.

**Table 1:** Patient baseline characteristics.

Characteristic	Group A1 (n=60)	Group A2 (n=60)	Group B1 (n=60)	Group B2 (n=60)	F/χ²	P
Age (years)	32.5 ± 7.8	33.1 ± 7.6	32.8 ± 7.9	33.0 ± 7.5	0.270	0.846
BMI (kg/m²)	23.5 ± 3.4	23.8 ± 3.3	23.7 ± 3.5	23.6 ± 3.4	0.150	0.930
Smoking history [n(%)]	15 (25.0%)	14 (23.3%)	16 (26.7%)	15 (25.0%)	0.170	0.982
Other HPV-related medical history [n(%)]	20 (33.3%)	18 (30.0%)	22 (36.7%)	19 (31.7%)	0.640	0.888
History of hysterectomy [n(%)]	5 (8.3%)	6 (10.0%)	5 (8.3%)	6 (10.0%)	0.150	0.982

### 3.2 Comparison of HPV positive rate and negative conversion rate

The positive rates of HPV in the four groups before treatment were 95,0% in group A1, 93.3% in group A2, 96.7% in group B1 and 95.0% in group B2, respectively, with no significant difference (P>0.05). At 3 and 6 months after treatment, the positive rate of HPV in all four groups decreased significantly, with the greatest decrease in group B2 (P<0.05). Moreover, HPV negative conversion rate was significantly increased at 3 and 6 months after treatment, with the highest negative conversion rate in group B2 (P<0.05), as shown in **Table 2**.

**Table 2:** Comparison of HPV positive rate and negative conversion rate [n(%)].

HPV status	Time point	Group A1 (n=60)	Group A2 (n=60)	Group B1 (n=60)	Group B2 (n=60)	χ²	P
HPV positive rate	Pre-treatment	57 (95.0%)	56 (93.3%)	58 (96.7%)	57 (95.0%)	0.286	0.962
	3 months later	31 (51.7%)	29 (48.3%)	30 (50.0%)	26 (43.3%)	8.274	0.041
	6 months later	20 (33.3%)	16 (26.7%)	19 (31.7%)	13 (21.7%)	9.351	0.025
HPV negative conver sion rate	3 months Later	29 (48.3%)	31 (51.7%)	30 (50.0%)	34 (56.7%)	7.238	0.045
	6 months later	40 (66.7%)	44 (73.3%)	41 (68.3%)	47 (78.3%)	10.842	0.012

### 3.3 Changes in specific HPV typing

At 3 and 6 months after treatment, the proportion of HPV16 and HPV18 in the four groups was significantly reduced, with the largest reduction in the B2 group. Other high-risk HPV subtypes, such as HPV31 and HPV33, were also significantly reduced after treatment (P<0.05), as shown in **Table 3**.

**Table 3:** Changes in specific HPV types [n(%)].

HPV subtype	Time points	Group A1 (n=60)	Group A2 (n=60)	Group B1 (n=60)	Group B2 (n=60)	χ²	P
HPV16	Pre-treatment	34 (56.7%)	35 (58.3%)	36 (60.0%)	36 (60.0%)	0.289	0.963
	3 months later	18 (30.0%)	17 (28.3%)	19 (31.7%)	15 (25.0%)	8.142	0.042
	6 months later	12 (20.0%)	9 (15.0%)	13 (21.7%)	8 (13.3%)	9.351	0.025
HPV18	Pre-treatment	20 (33.3%)	19 (31.7%)	21 (35.0%)	22 (36.7%)	0.221	0.974
	3 months later	10 (16.7%)	9 (15.0%)	11 (18.3%)	8 (13.3%)	7.829	0.049
	6 months later	6 (10.0%)	5 (8.3%)	7 (11.7%)	4 (6.7%)	8.914	0.037
HPV31	Pre-treatment	12 (20.0%)	11 (18.3%)	13 (21.7%)	12 (20.0%)	0.239	0.888
	3 months later	6 (10.0%)	5 (8.3%)	7 (11.7%)	4 (6.7%)	8.327	0.039
	6 months later	4 (6.7%)	3 (5.0%)	5 (8.3%)	2 (3.3%)	9.124	0.034
HPV33	Pre-treatment	9 (15.0%)	10 (16.7%)	8 (13.3%)	10 (16.7%)	0.223	0.973
	3 months later	5 (8.3%)	4 (6.7%)	6 (10.0%)	3 (5.0%)	7.715	0.046
	6 months later	3 (5.0%)	2 (3.3%)	4 (6.7%)	2 (3.3%)	8.823	0.040
Other high-risk types	Pre-treatment	8 (13.3%)	7 (11.7%)	9 (15.0%)	8 (13.3%)	0.241	0.969
	3 months later	4 (6.7%)	3 (5.0%)	5 (8.3%)	2 (3.3%)	7.926	0.050
	6 months later	2 (3.3%)	1 (1.7%)	3 (5.0%)	1 (1.7%)	8.944	0.036

### 3.4 Comparison of clinical efficacy

The cure rates were 58.3% in group A1, 68.3% in group A2, 65.0% in group B1 and 78.3% in group B2, respectively. The cure rate of B2 group was the highest (P<0.05), indicating that the combination of dressing for 5 hours and lighting for 40 minutes was the best treatment parameter. The recurrence rate was the lowest in B2 group (P<0.05), and there was no significant difference in progression rate among all groups (P>0.05), as shown in **Table 4**.

**Table 4:** Comparison of clinical efficacy [n(%)].

Outcome	Group A1	Group A2	Group B1	Group B2	χ²	P
	(n=60)	(n=60)	(n=60)	(n=60)		
Recovery rate	35 (58.3%)	41 (68.3%)	39 (65.0%)	47 (78.3%)	10.123	0.018
Recurrence rate	9 (15.0%)	6 (10.0%)	7 (11.7%)	4 (6.7%)	7.928	0.049
Progression rate	3 (5.0%)	2 (3.3%)	3 (5.0%)	1 (1.7%)	6.341	0.097

### 3.5 Incidence of adverse reactions

In terms of adverse reactions, increased vaginal discharge, spot-like bleeding, local pruritus and other symptoms occurred in all the four groups, but the overall incidence was low and there was no significant difference among the four groups (P>0.05). Cervical stenosis was mild and no serious cases were found, as shown in **Table 5**.

**Table 5:** Incidence of adverse reactions [n(%)].

Adverse reaction	Group A1 (n=60)	Group A2 (n=60)	Group B1 (n=60)	Group B2 (n=60)	χ²	P
Increased vaginal discharge	10 (16.7%)	8 (13.3%)	9 (15.0%)	6 (10.0%)	7.192	0.066
Punctate hemorrhage	8 (13.3%)	7 (11.7%)	7 (11.7%)	5 (8.3%)	6.492	0.090
Local itch discomfort	12 (20.0%)	10 (16.7%)	11 (18.3%)	8 (13.3%)	7.014	0.071
Cervical stenosis	3 (5.0%)	2 (3.3%)	3 (5.0%)	1 (1.7%)	5.238	0.155

### 3.6 Correlation analysis between clinical efficacy and HPV typing

**Table 6** shows the results of Pearson correlation analysis between HPV typing and clinical outcomes. The cure rates of HPV16 and HPV18 were positively correlated with the treatment effect, and the correlation coefficients were 0.432 (P = 0.003) and 0.421 (P = 0.004), respectively, indicating that these two high-risk HPV types responded well to ALA-PDT treatment. Recurrence and progression rates were inversely associated with all HPV subtypes.

**Table 6:** Correlation analysis between clinical efficacy and HPV typing.

HPV typing	Cure rate		Recurrence rate		Progression rate	
	r	P	r	P	r	P
HPV16	0.432	0.003	-0.289	0.045	-0.267	0.061
HPV18	0.421	0.004	-0.276	0.053	-0.254	0.070
HPV31	0.416	0.005	-0.264	0.062	-0.249	0.073
HPV33	0.408	0.006	-0.256	0.071	-0.242	0.078
Other high-risk types	0.401	0.007	-0.247	0.075	-0.235	0.082

## 4 Discussion

In recent years, clinical studies on photodynamic therapy (PDT) have involved a variety of diseases, including cancer and precancerous lesions, non-cancerous dysplasia, inflammation, capillary-hyperplastic diseases, and post-angioplasty stenosis, etc. (10). In the field of gynecology, PDT is used for condyloma acuminatum, high-risk cervical infection, vulva or cervical intraepithelial neoplasia and cervical carcinoma *in situ*. The use of a new generation of photosensitizers, such as 5-amino-ketovalerate (ALA), is safer and more effective in treating cervical HPV infection (14).

Foreign scholar Min Chul Choi et al. (15) conducted a retrospective study on 73 patients who received CIN II/III PDT treatment and found that the HPV eradication rates at 3 and 12 months after PDT treatment were 89.8% and 87.0%, respectively, indicating that PDT is an effective conservative treatment plan for young patients. Domestic scholar Li Rui et al. (16) randomly divided 86 patients with HR-HPV persistent cervical infection into two groups of conventional interferon treatment and ALA-PDT treatment. The results showed that the HPV negative conversion rate in the ALA-PDT group was significantly higher than that in the interferon group, confirming that ALA-PDT had significant effects in promoting HPV negative conversion, reducing the risk of cancer transformation and improving prognosis. Wang Li’s study (17) adopted Hela cell and HaCaT cell models and found that ALA-PDT could strongly inhibit proliferation and promote apoptosis of HPV-infected cells, but had little effect on normal cells, which further confirmed the safety and effectiveness of ALA-PDT. Based on the analysis of relevant literature and clinical data, Qiu Haixia et al. (18) concluded that PDT therapy with local and systematic photosensitizers can be used for the treatment of cervical epithelial neoplasia, among which local PDT therapy is mainly used for CIN- ii ~ 聂, and systematic PDT therapy is mainly used for CIN- ii ~ III. The effective rates of the two were 50%-95% and 88%-100%, respectively.

The objective of this study was to evaluate the efficacy and optimal therapeutic parameters of ALA-PDT in the treatment of HPV infection-associated CIN I. The results of the study showed that ALA-PDT was significantly effective in the treatment of HPV infective CIN I, especially in the combination of 5 hours of application and 40 minutes of illumination (group B2). Before treatment, there was no significant difference in the positive rate of HPV among all groups, but at 3 and 6 months after treatment, the positive rate of HPV decreased significantly in all groups, and the decrease was the largest in group B2, and the negative rate of HPV was also the highest. These results indicate that the antiviral effect of ALA-PDT can be enhanced by prolonging the time of application and illumination. ALA, as a photosensitizer, produces singlet oxygen and other reactive oxygen species by irradiating specific wavelengths of light, which can effectively destroy HPV-infected diseased cells. The extension of application time and light time may increase the absorption of photosensitizers and the production of reactive oxygen species, thus improving the therapeutic effect. This is consistent with the results of previous studies, such as one by Chen et al., which showed that ALA-PDT can significantly improve the cure rate of HPV-infected cervical intraepithelial neoplasia (CIN) and reduce the residual and recurrence rate of low-grade squamous intraepithelial lesions (LSIL). In a comparative study, HPV clearance and LSIL reversal rates were 79.0% and 80.6% in patients treated with ALA-PDT, compared with 62.3% and 64.2% in the control group (19).

In this study, HPV16 and HPV18 were the most common high-risk HPV subtypes. After treatment, the number of HPV16 and HPV18 decreased significantly in the four groups, with the largest reduction in the B2 group. Other high-risk HPV types, such as HPV31 and HPV33, were also significantly reduced after treatment. The significant reduction in high-risk HPV subtypes suggests that ALA-PDT has a strong ability to clear these subtypes, helping to reduce the risk of progression of CIN I. A study by Cang et al. pointed out that ALA-PDT had a higher clearance rate for HPV16 and HPV18 infections, in which 100% of HPV18 infections and 87.5% of HPV16 infections were cleared (20). Another study by Owczarek et al. found that in male patients with high-risk HPV infection, the HPV DNA clearance rate reached 66.67% after four ALA-PDT treatments (21).

B2 group had the highest cure rate and the lowest recurrence rate. This suggests that the combination of application for 5 hours and irradiation for 40 minutes is the best parameter for ALA-PDT treatment of HPV infection-associated CIN I. Long-term application and irradiation may increase the intracellular concentration of photosensitizers and the production of reactive oxygen species, and enhance the killing effect on diseased cells. The high cure rate and low recurrence rate in the B2 group suggest that this treatment regimen is not only effective in removing diseased cells, but also reduces the risk of recurrence.

In terms of adverse reactions, all groups had increased vaginal discharge, spot bleeding, local pruritus and other symptoms, but the overall incidence was low. Yokoyama et al. noted that common adverse effects of ALA-PDT include local pruritus, mild erythema, and pain. These reactions are generally brief and mild and do not have a significant impact on treatment outcomes (22). Another study by Wang et al. showed that the common adverse reactions in patients treated with ALA-PDT were mild burning sensation and local edema, which usually resolved within 24 hours (23).

This study found that HPV16 and HPV18 positive status was associated with better treatment outcomes, suggesting that these high-risk HPV subtypes responded favorably to ALA-PDT. Several recent clinical studies support subtype-specific differences in HPV clearance following ALA-PDT. For example, in HPV-positive women without cervical lesions, HPV16/18 infections were associated with significantly higher clearance rates compared with other high-risk types after ALA-PDT (100% for HPV18 and 87.5% for HPV16 vs 48.8% for other HR-HPV types at 6 months) (20). In patients with cervical low-grade squamous intraepithelial lesions treated with 5-ALA-mediated PDT, the HR-HPV clearance rate was higher in the HPV16/18 group than in the non-HPV16/18 group, although differences were not statistically significant at 6 months (12). The biological mechanisms underlying this observation are not fully understood but may relate to differences in lesion biology and viral activity. HPV16 and HPV18-infected epithelial cells exhibit higher metabolic activity and viral gene expression, which may enhance the uptake and intracellular accumulation of photosensitizers such as ALA, leading to increased generation of reactive oxygen species and more effective photodynamic cytotoxicity in infected cells. Additionally, PDT has been shown to exert immunomodulatory effects that may facilitate viral clearance and lesion regression, particularly in lesions with high viral loads. Despite these hypotheses, the association observed in the present study should be interpreted cautiously due to its retrospective design and moderate sample size. Further prospective studies with larger cohorts are needed to confirm subtype-specific responses to ALA-PDT and elucidate the underlying mechanisms.

Overall, the findings of this study suggest that ALA-PDT is a promising therapeutic approach for HPV-associated CIN I. The observed improvements in HPV clearance, clinical cure rates, and recurrence outcomes indicate that treatment efficacy may be influenced by both ALA application duration and light exposure time. Furthermore, the favorable response observed in patients with high-risk HPV subtypes provides additional evidence supporting the potential role of ALA-PDT in the management of HPV-related cervical lesions. Nevertheless, these findings should be interpreted in light of several methodological limitations, which are discussed below.

## 5 Clinical significance and limitations

This study demonstrates that ALA-PDT is an effective and safe therapeutic option for patients with HPV-associated CIN I. Among the treatment regimens evaluated, 5-hour ALA application combined with 40-minute light exposure achieved the highest cure rate and the lowest recurrence rate. High-risk HPV subtypes, particularly HPV16 and HPV18, showed favorable responses with substantial clearance rates following treatment. These findings suggest that ALA-PDT may represent a minimally invasive and fertility-preserving therapeutic strategy for patients with early cervical lesions and persistent HPV infection.

Several limitations should be acknowledged. First, this was a single-center study, which may limit the generalizability of the findings to broader patient populations. Second, the study did not include an untreated or placebo control group. Because CIN I is known to have a relatively high spontaneous regression rate, some of the observed clinical improvements may have been influenced by the natural course of the disease. The decision not to include an untreated control group was mainly based on ethical and clinical considerations, as all enrolled patients had confirmed HPV-associated CIN I and actively sought treatment after diagnosis. Therefore, prolonged observation without intervention was considered inappropriate in routine clinical practice. Nevertheless, the absence of a control group may have introduced potential selection and interpretation bias, and the treatment effect observed in this study should be interpreted with caution. Third, although patients were followed for up to 12 months, the efficacy outcomes were primarily evaluated within the first 6 months after treatment, and longer-term outcomes remain unclear. Finally, the sample size of 60 patients per group may have limited the statistical power of subgroup analyses, particularly those involving specific HPV subtype distributions and treatment-response correlations. Future multicenter prospective studies with larger sample sizes, longer follow-up periods, and appropriate control groups are warranted to further validate the efficacy of ALA-PDT and optimize treatment protocols.

## 6 Conclusion

ALA-PDT is a safe and effective treatment for HPV-associated CIN I. Among the tested regimens, a 5-hour ALA application combined with 40-minute light exposure provides the most favorable therapeutic outcomes, including the highest cure rate, lowest recurrence rate, and significant clearance of high-risk HPV subtypes. These results support the use of ALA-PDT as a minimally invasive, fertility-preserving treatment option for early cervical lesions. Future studies should focus on multicenter trials, longer follow-up durations, and optimization of treatment parameters to further enhance clinical efficacy and safety.

## Data Availability

The original contributions presented in the study are included in the article/supplementary material. Further inquiries can be directed to the corresponding author.
